# Does foetal gender influence maternal thyroid parameters in pregnancy?

**DOI:** 10.1530/ETJ-21-0001

**Published:** 2021-06-30

**Authors:** Georgiana Sitoris, Flora Veltri, Pierre Kleynen, Malika Ichiche, Serge Rozenberg, Kris G Poppe

**Affiliations:** 1 Endocrine Unit Centre Hospitalier Universitaire (CHU) Saint-Pierre, Université Libre de Bruxelles (ULB), Brussels, Belgium; 2 Department of Gynecology and Obstetrics, Centre Hospitalier Universitaire (CHU) Saint-Pierre, Université Libre de Bruxelles (ULB), Brussels, Belgium

**Keywords:** foetal, reference ranges, pregnancy, thyroid dysfunction, thyroid autoimmunity

## Abstract

**Objective:**

It is unknown if foetal gender influences maternal thyroid function during pregnancy. We therefore investigated the prevalence of thyroid disorders and determined first-trimester TSH reference ranges according to gender.

**Methods:**

A cross-sectional study involving 1663 women with an ongoing pregnancy was conducted. Twin and assisted pregnancies and l-thyroxine or antithyroid treatment before pregnancy were exclusion criteria. Serum TSH, free T4 (FT4) and thyroid peroxidase antibodies (TPOAb) were measured at median (interquartile range; IQR) 13 (11–17) weeks of gestation. Subclinical hypothyroidism (SCH) was present when serum TSH levels were >3.74 mIU/L with normal FT4 levels (10.29–18.02 pmol/L), and thyroid autoimmunity (TAI) was present when TPOAb were ≥60 kIU/L.

**Results:**

Eight hundred and forty-seven women were pregnant with a female foetus (FF) and 816 with a male foetus (MF). In women without TAI and during the gestational age period between 9 and 13 weeks (with presumed high-serum hCG levels), median (IQR range) serum TSH in the FF group was lower than that in the MF group: 1.13 (0.72–1.74) vs 1.24 (0.71–1.98) mIU/L; *P* = 0.021. First-trimester gender-specific TSH reference range was 0.03–3.53 mIU/L in the FF group and 0.03–3.89 mIU/L in the MF group. The prevalence of SCH and TAI was comparable between the FF and MF group: 4.4% vs 5.4%; *P* = 0.345 and 4.9% vs 7.5%; *P* = 0.079, respectively.

**Conclusions:**

Women pregnant with an MF have slightly but significantly higher TSH levels and a higher upper limit of the first-trimester TSH reference range, compared with pregnancies with a FF. We hypothesise that this difference may be related to higher hCG levels in women pregnant with a FF, although we were unable to measure hCG in this study. Further studies are required to investigate if this difference has any clinical relevance.

## Introduction

Thyroid dysfunction in pregnant women is mainly caused by the presence of thyroid autoimmunity (TAI), reflected by increased thyroperoxidase (TPO) and/or thyroglobulin (Tg) antibody levels (1, 2). Over the years, a number of other variables and conditions have been shown to alter serum TSH levels (3). These include demographic characteristics (age, BMI and ethnic background), environmental factors (tobacco and endocrine disruptors), the obstetric history (parity), nutritional factors (iodine and iron), high estradiol levels (ovarian stimulation), high hCG levels (twin pregnancies, during gestational weeks 9–13) and, finally, the variability in the TSH assays (1, 2, 3, 4, 5, 6).

In a number of studies, the impact of some thyroid parameters on pregnancy outcomes was different according to the foetal gender (7, 8, 9). In the study by Zhang *et al.*, the lowest percentiles of maternal free T4 (FT4) were associated with a higher birth weight in case of a male foetus (MF) only (8). In another study, early maternal FT4 levels in euthyroid women were inversely associated with birth weight with a stronger association in case of an MF (7). Finally, among euthyroid women with TAI, after adjustment for confounders, only women pregnant with a female foetus (FF) had an increased risk of preterm births (9). Differences in these pregnancy outcomes according to the foetal gender could be explained in part by a sex-specific maternal–placental–foetal interaction such as higher serum hCG levels in women expecting a FF (10, 11).

It is, however, not well established, whether the prevalence of thyroid disorders in pregnant women is different according to the foetal gender. Additionally, foetal gender-specific reference range has not been established yet. Actually, for the determination of TSH pregnancy-specific reference range, the European Thyroid Association and American Thyroid Association (ATA) guidelines propose to exclude the following conditions: TAI, severe iodine deficiency, twin and assisted pregnancies and using local/institutional assay for TSH (12, 13).

Therefore, the aims of this study were to investigate the prevalence of thyroid dysfunction and autoimmunity in pregnant women according to the foetal gender and to determine first-trimester gender-specific reference ranges.

## Materials and methods

### Overall study design

The obstetric clinic of the Centre Hospitalier Universitaire Saint-Pierre is a downtown, public university maternity centre in Brussels, Belgium. In our centre, during the first antenatal consultation, demographic and obstetrical data are noted and systematically completed with biological analyses including TSH, FT4 and thyroid peroxidase antibodies (TPOAb) measurements. The ethnic background of the women is based on a history taken by the social workers that includes systematically the nationality at birth and the origin of the women. Gestational age is established using ultrasound, expressed in full weeks from the first day of the last menstrual period. Data on foetal genders were collected retrospectively (it can be determined accurately only from the second foetal ultrasound that takes place at ~18th to 20th week of gestation).

We report here a cross-sectional analysis of women with ongoing pregnancies (period 2 January 2013/31 December 2014) that was nested within the ongoing prospective collection of women’s obstetrical parameters and biological data. After the exclusion of pregnancies resulting from assisted reproduction (*n* = 32), multiple pregnancies (*n* = 48) and women treated with l-thyroxine or antithyroid drugs before screening (*n* = 39), 1663 women were included for comparison of the prevalence of thyroid disorders and baseline/obstetric characteristics between women pregnant with an FF (*n* = 847) or a MF (*n* = 816).

**Figure 1:**
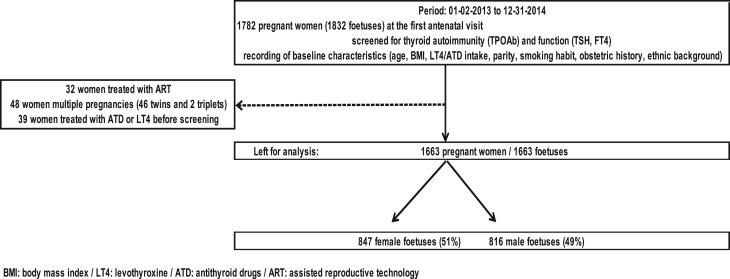
Flowchart of the study selection process.

In Fig. 1, we illustrate the study selection process in a flowchart.

For the upper limit of serum TSH during the first trimester of pregnancy, we used our institutional cut-off level (3.74 mIU/L), and for the lower limit of FT4, we used 10.29 pmol/L, based on a previous study of our group (14). Subclinical hypothyroidism (SCH) was defined as a serum TSH level >3.74 mIU/L and subclinical hyperthyroidism was defined as a serum TSH level <0.06 mIU/L together with a normal FT4 level (10.29–18.02 pmol/L), respectively.

Isolated hypothyroxinaemia (IH) was defined as an FT4 level <2.5th percentile (10.29 pmol/L) with a normal serum TSH (0.06–3.74 mIU/L). Finally, TAI was present when TPOAb levels were ≥60 kIU/L.

Following the recommendations of the International Federation of Clinical Chemistry on the calculation of TSH reference ranges, and the specific ATA guidelines on TSH reference ranges during pregnancy, women with TAI, twin and assisted pregnancies and outlier values were excluded (13, 15). For the outliers, we applied the formulae of Hoaglin (upper limit = Q3 + (2.2 × [Q3−Q1]), lower limit = Q1 − (2.2 × [Q3−Q1])); Q = Quartile (16). Once the upper and lower limits were obtained, results higher than the upper limit and lower than the lower limit were removed from the data (*n* = 13 in the upper levels). We did not correct for severe iodine deficiency in our study, but we know from a previous study in the Brussels metropolitan area (including our centre) that during the first trimester of pregnancy, the median (IQR) urinary iodine is 117 (70–189) µg/L (17).

The study was approved by the institutional review board (AK/15-11-114/4568).

### Serum assay

All provisions were implemented by the laboratory of hormonology of our institution. Serum TSH, FT4 and TPOAb levels were measured using the Chemiluminescence Centaur XP Siemens immunoanalyzer. The reference values for non-pregnant women were 0.3–4.0 mIU/L, 10.3–25.7 pmol/L (0.8–2.0 ng/dL) and <60 kIU/L for TSH, FT4 and TPOAb, respectively. The total imprecision CVs were 6.9, 4.2 and 7.6% for TSH, FT4 and TPOAb, respectively. For conversion of FT4, 1 ng/dL = 12.9 pmol/L.

### Statistical analysis

Data were stored in a Microsoft Excel database, and statistical analyses were performed using Stata 11.2 software (Lakeway drive, Texas, USA). Continuous data are expressed as median (interquartile range; IQR range) when not normally distributed and as mean ± SD for normally distributed data. Categorical data are presented as numbers (percentage) of cases. Differences between all groups were analysed by Fisher’s exact tests for categorical data and by a *t*-test for continuous data.

For Table 1, because foetal gender cannot be significantly associated with any outcomes because of Mendelian randomisation, no *P* values are given.

**Table 1:** Demographic and obstetric parameters in all women and according to the foetal gender.

Pregnancy and obstetrical data	All women	Female foetus	Male foetus
Continuous data^a^			
Categorical data (*n* (%))	*n* = 1663	*n* = 847 (51%)	*n* = 816 (49%)
Maternal age (years)	29.9 ± 5.8	29.7 ± 5.9	30.1 ± 5.8
High maternal (age ≥35 years)	389 (23.3%)	197 (23.3%)	192 (23.5%)
Caucasian background	392 (23.6%)	197 (23.3%)	195 (23.9%)
BMI pre-pregnancy (kg/m^2^)	25.6 ± 4.8	25.5 ± 4.9	25.8 ± 4.7
Obesity (BMI ≥ 30 kg/m^2^)	267 (16.1%)	129 (15.2%)	138 (16.9%)
Tobacco use	252 (15.2%)	130 (15.3%)	122 (15.0%)
Parity (*n*)	1 (0–2)	1 (0–2)	1 (0–2)
Multiparty (≥3)	203 (12.2%)	106 (12.5%)	97 (11.9%)
History of >1 first-trimester MC	111 (6.7%)	55 (6.5%)	56 (6.9%)

No *P* values are given because foetal gender cannot be significantly associated with any outcomes because of Mendelian randomisation.

^a^Continuous data are expressed as mean ± SD or median (IQR range).

MC, miscarriage.

For the logistic regression analyses, dependent outcomes were: increased serum TSH levels, TAI and IH, respectively. Besides the foetal gender, other independent variables were gestational age at blood sampling, maternal age, BMI, a Caucasian background, tobacco use, parity, a history of >1 first-trimester miscarriage and TAI.

When one or more independent variables were associated with a dependent outcome in the univariable analysis, they were further implemented in a multivariable logistic analysis. We did not make a *P*-value correction for the logistic regression as we performed separate analyses for each outcome. For the multiple comparisons between groups with and without TAI, according to the foetal gender and gestational age, we performed a correction and considered *P* < 0.01 as significant.

All other results were considered significant whenever *P* < 0.05.

## Results

Table 1 shows demographic and obstetric parameters in all women and according to the foetal gender.

In total, 1663 pregnant women were included of whom 847 were with a FF (51%) and 816 were with an MF (49%); *P* = 0.282. Baseline characteristics (maternal age, BMI before pregnancy and prevalence of women with other than Caucasian background), obstetric history data (parity and history of >1 miscarriage) and tobacco use are shown (no *P* values are given, see the ‘Statistical analysis’ section).

Table 2 shows thyroid function and autoimmune parameters in all women and according to the foetal gender.

**Table 2:** Thyroid function and autoimmune parameters in all women and according to the foetal gender.

Gestational age and thyroid function/TAI data	All women	Female foetus	Male foetus	*P*
Continuous data^a^				
Categorical data (*n* (%))	*n* = 1663	*n* = 847 (51%)	*n* = 816 (49%)	
Gestational age at blood sampling (weeks)	13 (11–17)	13 (11–17)	13 (11–17)	
Gestational age at blood sampling (<9 >13 weeks)	880 (52.9%)	449 (53.0%)	431 (52.8%)	
TSH (mIU/L)	1.44 (0.89–2.10)	1.40 (0.89–2.04)	1.49 (0.89–2.18)	**0.022**
<0.06 mIU/L	42 (2.5%)	26 (3.1%)	16 (2.0%)	0.149
0.06–2.50 mIU/L	1335 (80.3%)	693 (81.8%)	642 (78.7%)	0.108
2.51–3.74 mIU/L	203 (12.2%)	90 (10.6%)	113 (13.8%)	**0.045**
>3.74 mIU/L	83 (5.0%)	38 (4.5%)	45 (5.5%)	0.336
FT4 (pmol/L)^b^	14.2 (12.9–15.4)	14.2 (12.9–15.4)	12.9 (12.9–15.4)	0.708
Isolated hypothyroxinaemia^b^/^c^	11 (0.7%)	4 (0.5%)	7 (0.9%)	0.337
TPOAb (kIU/L)	28 (28–38)	28 (28–37)	29 (28–38)	0.228
TAI (TPOAb ≥ 60 kIU/L)	116 (7.0%)	50 (5.9%)	66 (8.1%)	0.079

Bold indicates statistical significance.

^a^Continuous data are expressed as median (IQR range); ^b^calculated on 1646; ^c^normal TSH level and FT4 < 10.29 pmol/L.

TSH, thyrotropin; FT4, free thyroxine; TPOAb, thyroid peroxidase autoantibodies; TAI, thyroid autoimmunity.

Gestational age at blood sampling (median (IQR)) was comparable between both groups (13 (11–17)) weeks; *P* = 0.699).

Median (IQR) serum TSH level for all women was 1.44 (0.89–2.10) mIU/L; levels were significantly lower in the FF group compared with the MF group: 1.40 (0.89–2.04) vs 1.49 (0.89–2.18) mIU/L; *P* = 0.022. Forty-two women had suppressed TSH levels (prevalence: 2.5%). The prevalence of women with high-normal TSH levels (2.51–3.74 mIU/L) was lower in the FF group compared with the MF group: 10.6% vs 13.8%; *P* = 0.045. The prevalence of women with increased TSH levels was comparable between the FF and MF group: 4.5% vs 5.5%; *P* = 0.708.

Median (IQR) serum FT4 level for all women was 14.2 (12.9–15.4) pmol/L and comparable between FF and MF group: 14.2 (12.9–15.4) vs 12.9 (12.9–15.4) pmol/L; *P* = 0.708. Eleven women had IH (prevalence: 0.7%) without a difference between both groups; four women in the FF group vs seven in the MF group; *P* = 0.337.

The prevalence of TAI was 7% (*n* = 116) and comparable between both groups: 5.9% in FF vs 8.1% in MF; *P* = 0.079.

Other results on thyroid dysfunction and reference ranges are not shown in Table 2.

Seventeen women had overt hyperthyroidism (prevalence: 1.0%); eleven (1.3%) in the FF vs six (0.7%) in the MF group; *P* = 0.249. Twenty-five women had subclinical hyperthyroidism (prevalence: 1.5%); 15 (1.8%) in the FF vs 10 (1.2%) in the MF group; *P* = 0.353. Of all women with suppressed serum TSH, 9.5% had TAI; 7.7% in the FF vs 12.5% in the MF group; *P* = 0.641.

Most (83.3%) women with suppressed TSH levels (*n* = 42) were detected during the first trimester.

One woman in the FF group had overt hypothyroidism (prevalence: 0.12%) and none in the MF group; overall prevalence: 0.06%. Eighty-one women had SCH (overall prevalence: 4.9%); 37 (4.4%) in the FF vs 44 (5.4%) in the MF group; *P* = 0.345. Among women with SCH, 13.5% had TAI; 16.2% in the FF vs 11.4% TAI in the MF group; *P* = 0.525.

After the exclusion of 13 outliers in the upper range, first-trimester serum TSH reference range (median gestational age 11 (9–12) weeks) for the whole cohort was (0.03–3.67) mIU/L (702 women). In the FF group (*n* = 360), it was (0.03–3.53) mIU/L, and in the MF group (*n* = 342), it was (0.03–3.89) mIU/L. Gestational ages were comparable between groups: 11 (9–12) in FF vs 11 (9–12) weeks in MF; *P* = 0.400.

First-trimester FT4 reference range for the whole cohort was (10.30–18.02) pmol/L and was similar in the two study groups.

**Figure 2:**
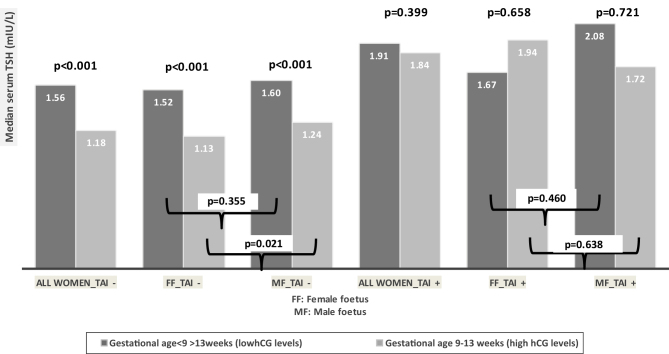
Serum TSH levels in women with and without thyroid autoimmunity and according to gestational age <9 and >13 weeks.

Figure 2 shows serum TSH levels in women with and without TAI and according to gestational age <9 and >13 weeks.

In women without TAI, during the period of presumed low(er) hCG levels (median 17 (14–22) weeks), median TSH was comparable between the FF and MF group: 1.52 (1.02–2.19) vs 1.60 (0.98–2.21) mIU/L; *P* = 0.355. During the period of presumed high(er) hCG levels (median 12 (11–12) weeks), median TSH was lower in the FF vs the MF group: 1.13 (0.72–1.74) vs 1.24 (0.71–1.98) mIU/L; *P* = 0.021.

In women with TAI, during the period of presumed low(er) hCG levels (median 15 (8–22) weeks), median TSH was comparable between the FF and MF group: 1.67 (1.20–3.13) vs 2.08 (1.44–2.62) mIU/L; *P* = 0.460. During the period of presumed high(er) hCG levels (median 12 (11–12) weeks), median TSH was comparable between the FF and MF group: 1.94 (1.29–2.56) vs 1.72 (1.24–2.82) mIU/L; *P* = 0.638.

Table 3 shows the results of the univariable and multivariable logistic regression analyses with TSH >3.74 mIU/L, TAI and IH as dependent outcomes.

**Table 3:** Univariable and multivariable logistic regression analysis with TSH >3.74 mIU/L, TAI and IH as dependent outcomes.

Independent variables	Univariable analysis						Multivariable analysis			
	Dependent outcomes						Dependent outcomes			
	TSH >3.74 mIU/L		TAI		IH		TSH >3.74 mIU/L		TAI	
	OR (95% CI)	*P*	OR (95% CI)	*P*	OR (95% CI)	*P*	aOR (95% CI)	*P*	aOR (95% CI)	*P*
Female foetus	0.81 (0.52–1.25)	0.337	0.71 (0.49–1.04)	0.082	0.55 (0.16–1.89)	0.344				
*Male foetus*	*1.24 (0.80*–*1.94)*	*0.337*	*1.40 (0.96*–*2.05)*	*0.082*	*1.81 (0.53*–*6.22)*	*0.344*				
GA @ blood sampling (<9 >13 weeks)	1.37 (0.87–2.15)	0.173	/		1.07 (0.33–3.51)	0.914				
Maternal age (years)	0.98 (0.94–1.02)	0.317	1.05 (1.01–1.08)	**0.008**	1.08 (0.98–1.20)	0.124			1.05 (1.02–1.09)	**0.001**
BMI (kg/m^2^)	1.01 (0.96–1.05)	0.744	0.95 (0.91–0.99)	**0.022**	1.07 (0.97–1.18)	0.201			0.95 (0.90–0.99)	**0.015**
Caucasian background	1.52 (0.94–2.44)	0.090	1.64 (1.10–2.50)	**0.017**	0.73 (0.16–3.38)	0.684			1.60 (1.06–2.41)	**0.027**
Smoking (yes/no)	1.59 (0.93–2.73)	0.091	1.51 (0.94–2.41)	0.087	0.56 (0.07–4.41)	0.584				
Parity (*n*)	0.73 (0.58–0.92)	**0.007**	0.93 (0.79–1.10)	0.406	1.43 (0.98–2.09)	0.063	0.74 (0.59–0.92)	0.080		
TAI	2.40 (1.26–4.56)	**0.008**	/		5.10 (1.33–19.49)	**0.017**	2.34 (1.23–4.47)	**0.010**		

Results are given as odds ratios (OR) (95% CI); *P* values. Bold indicates statistical significance.

FT4, free thyroxine; GA, gestational age; IH, isolated hypothyroxinaemia (TSH level <3.74 mIU/L and FT4 <10.29 pmol/L); TAI, thyroid autoimmunity (TPOAb ≥ 60 kIU/L); TPOAb, thyroid peroxidase autoantibodies; TSH, thyrotropin.

In the univariable analysis, TSH >3.74 mIU/L was associated with parity (OR 0.73 (95% CI 0.58–0.92)); *P* = 0.007 and TAI (OR 2.40 (95% CI 1.26–4.56)); *P* = 0.008. In the multivariable analysis, only the association with TAI persisted (OR 2.34 (95% CI 1.23–4.47)); *P* = 0.010.

Associations with TAI were present with maternal age (OR 1.05 (95% CI 1.01–1.08)); *P* = 0.008, BMI (OR 0.95 (95% CI 0.91–0.99)); *P* = 0.022 and a Caucasian background (OR 1.64 (95% CI 1.10–2.50)); *P* = 0.017. All these associations persisted in the multivariable analysis.

TAI was the only variable associated with IH (OR 5.10 (95% CI 1.33–19.49)); *P* = 0.017.

## Discussion

The main observation of this study was the significantly higher median serum TSH level and higher upper limit of first-trimester gender-specific TSH reference range in women pregnant with an MF vs a FF.

We hypothesise that this is associated with the higher hCG levels in women pregnant with a FF, as reported in a number of papers (10, 18, 19, 20). Furthermore, different isoforms of hCG with another affinity/action on the TSH receptor have been described (21, 22, 23). Finally, it is suggested that concentrations of the antiangiogenic soluble fms-like tyrosine kinase (sFlt1) and proangiogenic placental growth factor (PlGF) vary according to the foetal gender and may partially influence the highly vascularised thyroid through the hCG stimulation response (24, 25). Nevertheless, differences in thyroid function according to the foetal gender seem to persist after adjustment for sFlt1 and PlGF (26).

hCG is a glycoprotein hormone with an identical α-subunit as TSH, and the hormone specificity was determined by the β-subunit (26). The thyrotropic activity of 1 IU hCG is equivalent to 0.5–0.8 mIU/L TSH (27, 28). Glinoer *et al.* were the first to report the association between hCG concentrations and thyroid function during pregnancy (29). More than 50 years ago, Brody and Carstroem described higher serum hCG levels in women pregnant with a FF during the third trimester (30). Meanwhile, a number of groups reported similar results during all trimesters (11, 18, 31). Moreover, in a study in an *in vitro* fertilisation setting, already 3 weeks after the embryo implantation, hCG levels were 18.5% higher in pregnancies with a FF (19). Korevaar *et al.* reported mean hCG levels of 41.825 IU/L in pregnancies with a FF compared with 38.363 IU/L in case of an MF (*P* < 0.001), in 14.4 weeks pregnant women, after adjusting results for gestational age at blood sampling, maternal age, parity, smoking, ethnicity and BMI (26). The higher hCG levels in FF seem to be ethnicity dependent with a greater ratio in White (13%) vs Asian women (9%) in one study, and in women with a Moroccan, Turkish and Surinamese background vs Caucasian women in another one (18, 26). In our study, a Caucasian background was not associated with thyroid dysfunction.

Moreover, another argument in favour of the hCG hypothesis in our study is the fact that median TSH levels were comparable between both groups in women during the period <9 and >13 weeks of gestation (the period with presumed low(er) hCG levels). Similarly, Korevaar *et al.* reported comparable serum TSH and FT4 levels when hCG levels were in the low ranges (23). Furthermore, when we compared serum TSH levels during the period of (presumed) high-serum hCG levels (9–13 weeks of gestational age), they were indeed significantly different according to the foetal gender. However, in the study by Korevaar *et al.*, even when comparable high hCG levels were taken into account, differences in thyroidal hormone response between women carrying a FF vs an MF were noted (23). This means that some other factors (such as TAI) also play a role in the response of the thyroid gland to serum hCG levels. Indeed, also in our study, in women with TAI and in the period of presumed high(er) hCG levels, we did not observe a difference in serum TSH levels between both study groups. These features add strength to the fact that the presence of TAI is the variable with the strongest impact on thyroid function and are in line with previous study results (1, 3, 32).

Foetal gender was not associated with increased serum TSH levels (and SCH), but TAI (higher risk) and parity (lower risk) were. Our results are consistent with those in literature concerning TAI but not for parity (3).

Concerning our second main study result, the difference in the TSH upper limit of gender-specific reference range during the first trimester, this should be investigated in relation to pregnancy outcomes to know if it has any clinical importance. Therefore, the foetal gender should be known before thyroid tests are measured. Actually, the first foetal ultrasound is performed too soon during pregnancy (~12 weeks) to determine the foetal gender with high accuracy, which is now ~79% (33). An option to determine foetal gender sooner is the non-invasive prenatal testing, a method based on the analysis of cell-free foetal DNA found in maternal blood early in pregnancy. This test has become a regular screening test for the most common foetal aneuploidies and X-linked disorders (34). Based on it, foetal gender can be determined with a high accuracy from the 7th week of gestation onwards. However, for the time being, the use of these technologies for sex selection raises a number of ethical issues (34).

Concerning the other results in the logistic regression analyses, foetal gender was not associated with the presence of TAI. Maternal age and a Caucasian background were associated with a higher risk and BMI with a lower risk of TAI. In a Danish study, maternal age >30 years was a risk factor for the development of all types of thyroid disease before, during and/or after pregnancy (35), but this was not the case in two other studies (36, 37). The association between women with a Caucasian background and a higher prevalence of TAI remains controversial in the literature, and mechanisms were discussed in a previous review paper (3). Obesity increases the susceptibility to harbour TAI with leptin as a peripheral determinant (38). In a study investigating predictors of TAI, a sensitivity analysis yielded a poor discriminative ability for TAI (2).

Finally, we observed no association between the foetal gender and IH; only TAI was associated. In a review paper, TAI was not an important etiologic factor for the development of IH (39). However, in a recent Dutch study, the prevalence of elevated TPOAb was twice as high in women with IH (taking all FT4 cut-offs into account), compared with that in euthyroid women (40). The role of iron deficiency needs to be investigated further in this context since it might link TAI and lower FT4 levels (1).

The major limitation of our study is the absence of hCG levels that would have consolidated our results. As a surrogate measurement, we used groups of the gestational age at blood sampling. Concerning the gender-specific reference ranges, we did not correct for (severe) iodine deficiency, but it has to be mentioned that in two studies serum TSH concentrations did not vary significantly according to the iodine status (lowest values <100 µg/L and highest >250 µg/L) (17, 41). This suggests that normal reference ranges can be determined based on data also from mildly iodine-deficient populations as it is the case in the Brussels area (17, 41).

The main strength of our study is the original observation on the difference in thyroid function/thyroid reference ranges according to the foetal gender.

In conclusion, women pregnant with an MF have higher serum TSH levels and a higher TSH upper limit of the first-trimester reference range compared with those in pregnancies with a FF. We hypothesise that this difference may be related to higher hCG levels in women pregnant with a FF, although we were unable to measure hCG in this study.

Further studies are needed to investigate whether gender-specific reference ranges have an impact on pregnancy outcomes, and therefore, foetal gender should be determined earlier during pregnancy.

## Declaration of interest

Kris G Poppe had no conflict of interest in relation to the current study but received in the period 2018–2020 lecture fees from the Berlin-Chemie, Merck and IBSA company. Georgiana Sitoris, Flora Veltri, Pierre Kleynen, Malika Ichiche and Serge Rozenberg had no conflict of interest.

## Funding

This work did not receive any specific grant from any funding agency in the public, commercial or not-for-profit sector.

## Author contribution statement

G S revised the manuscript. F V collected data and revised the manuscript. P K revised the manuscript. M I revised the manuscript. S R revised the manuscript and approved the final version. K G P designed and performed the study, acquired and analysed the data, drafted and revised the manuscript, and approved the final version.
